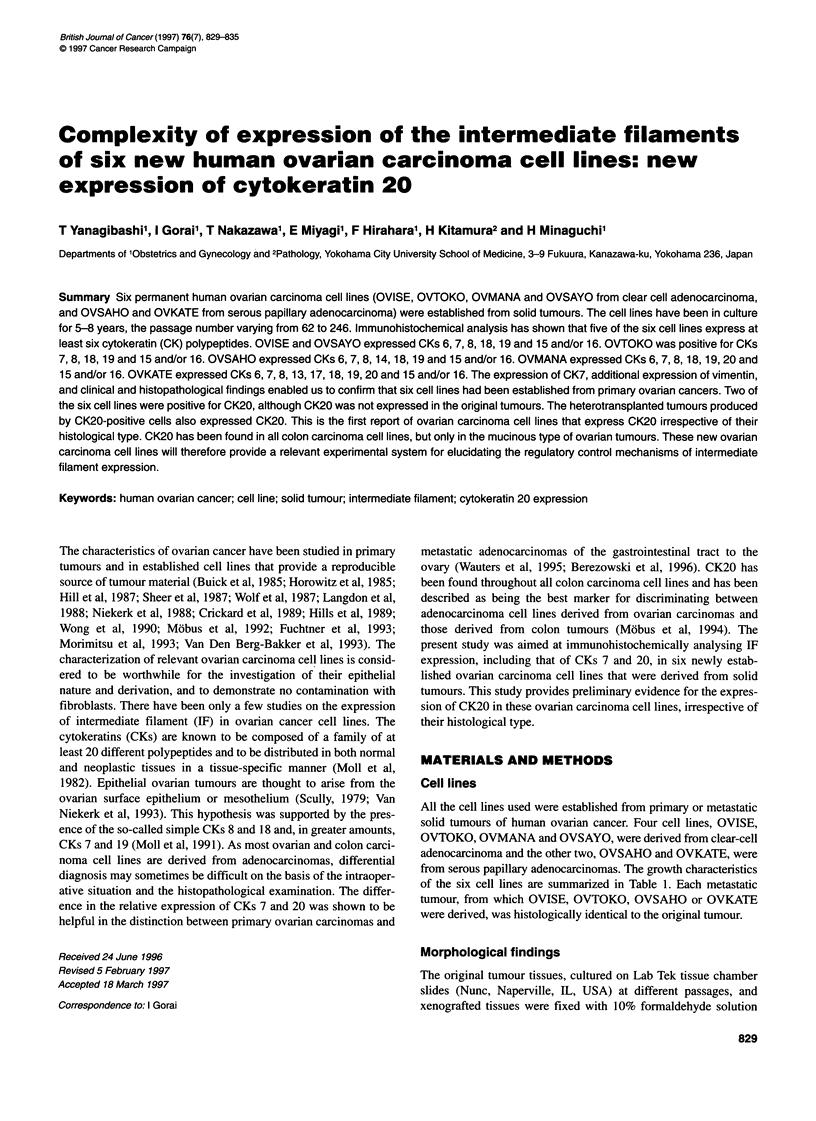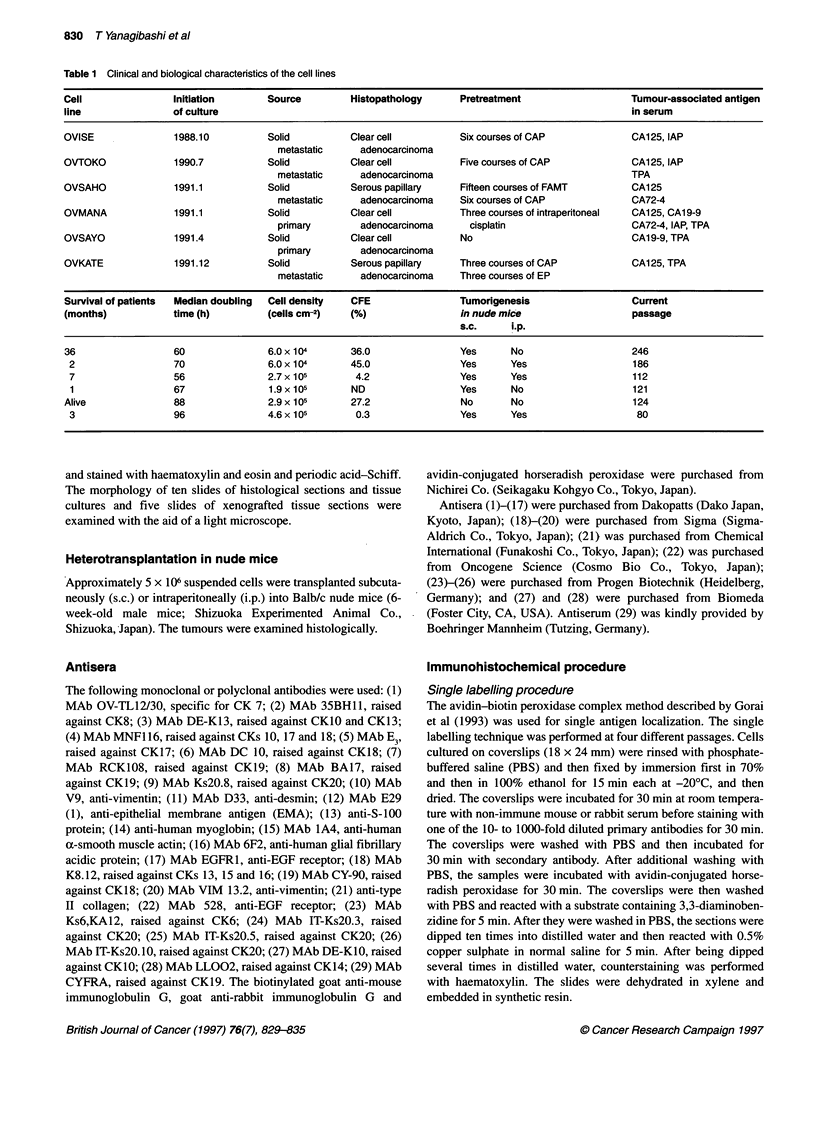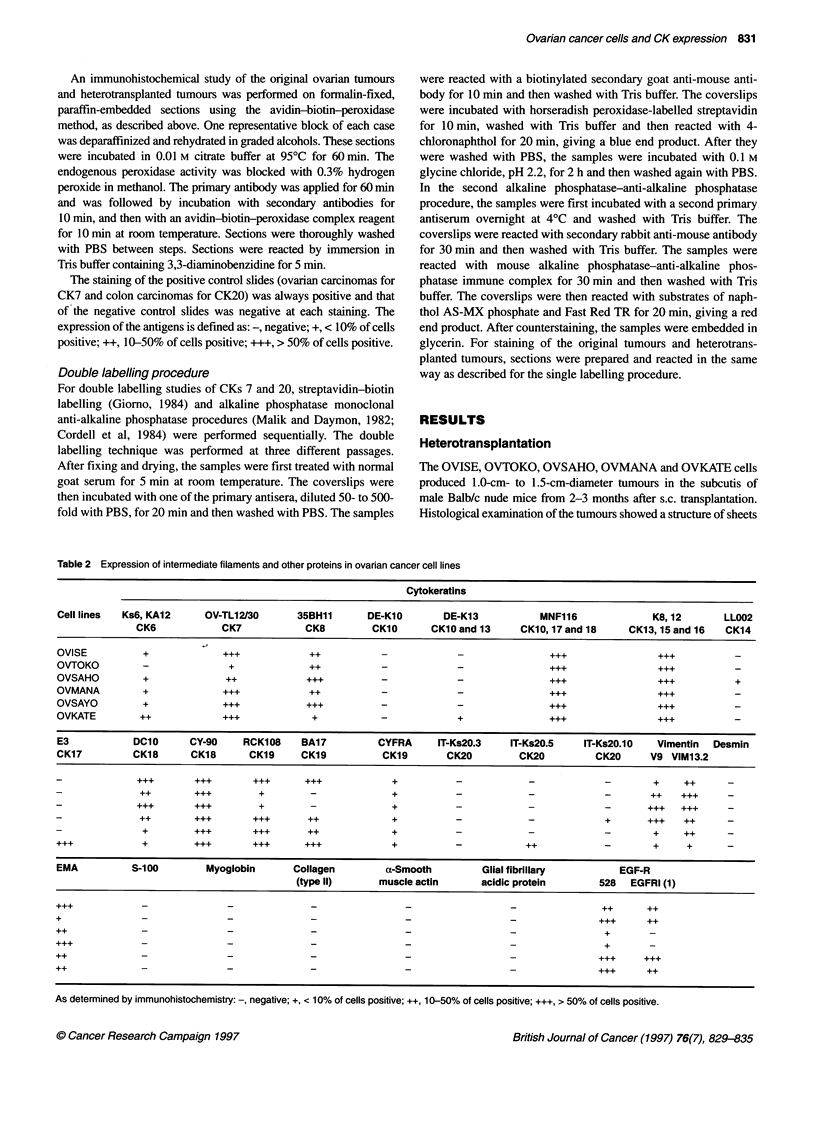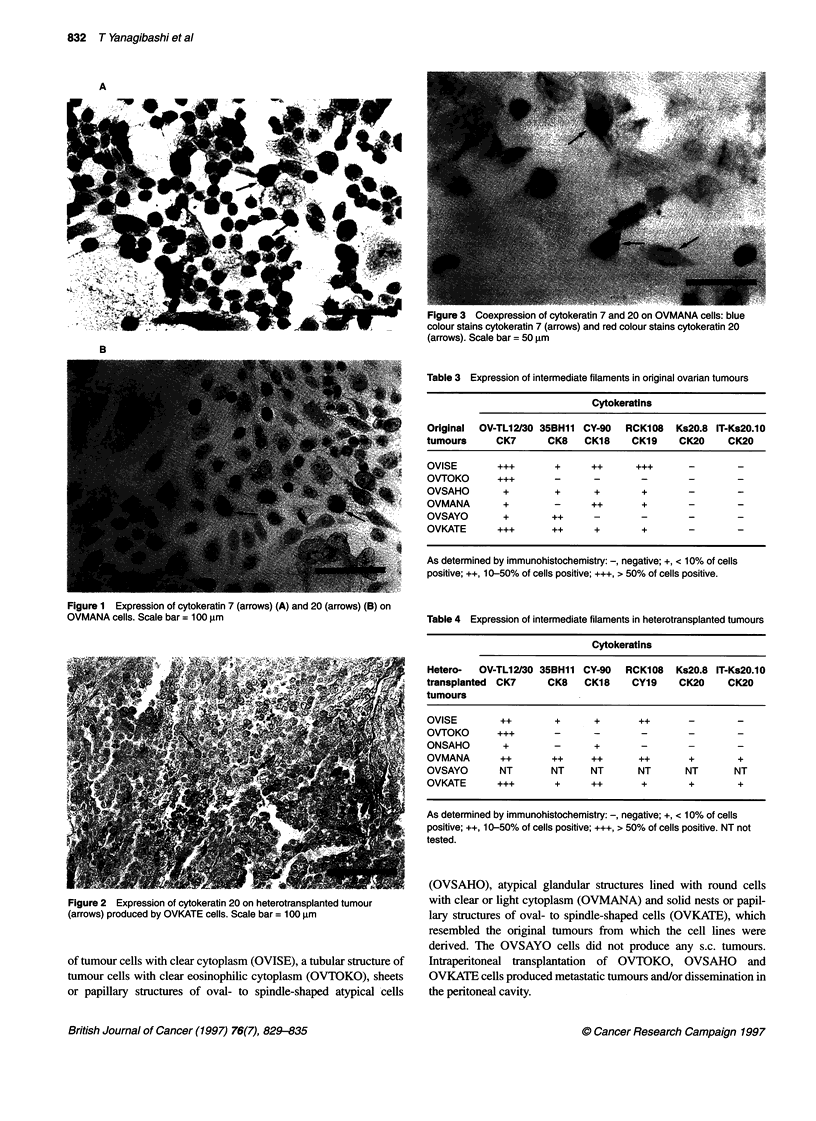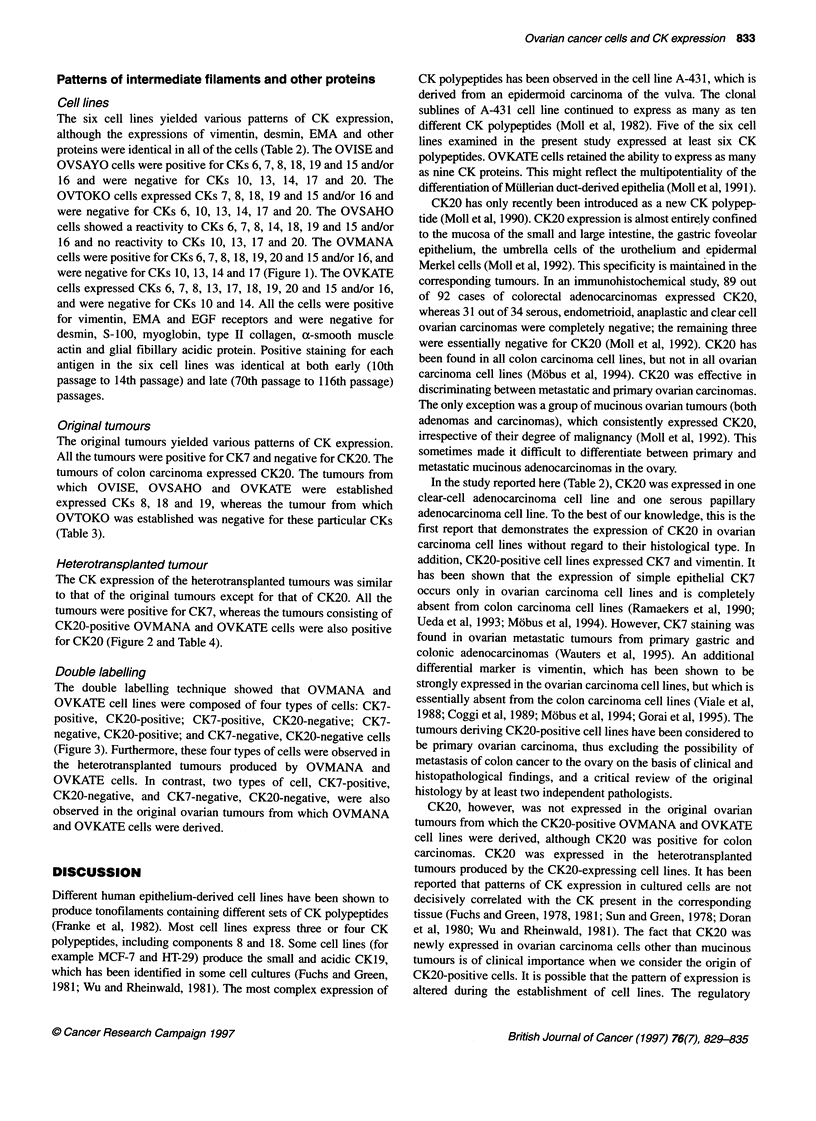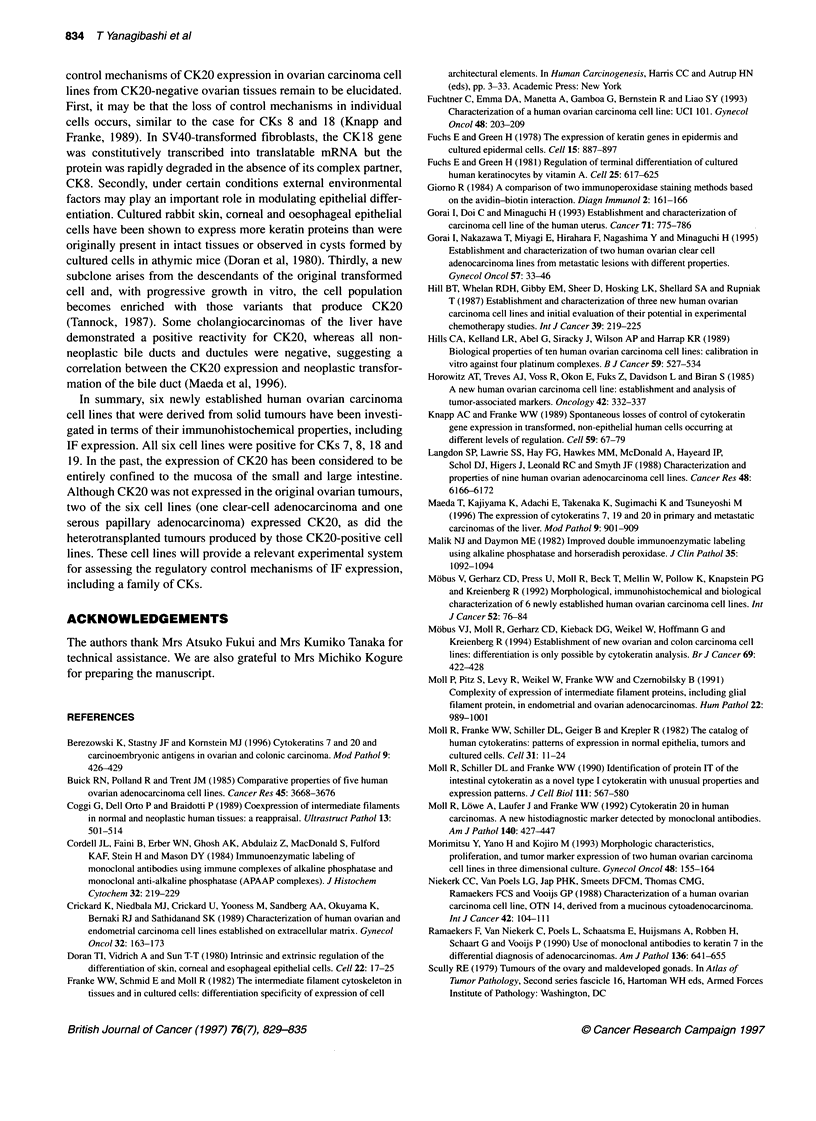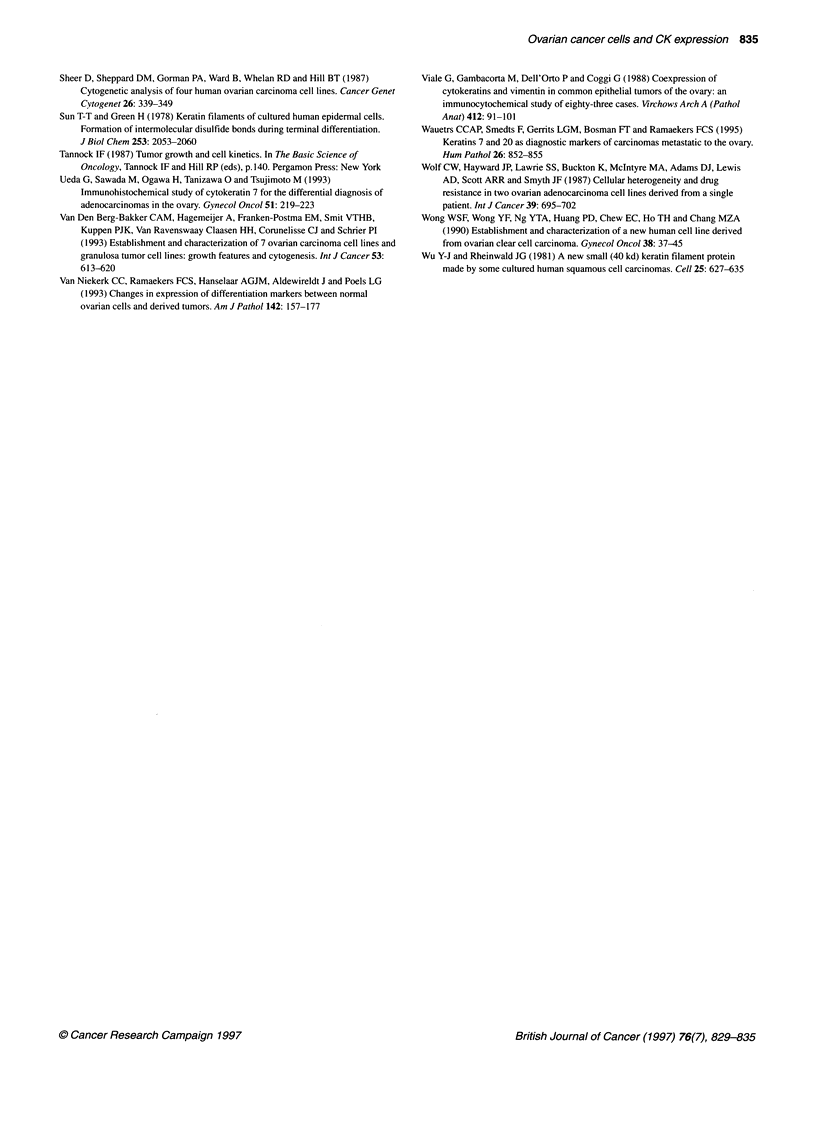# Complexity of expression of the intermediate filaments of six new human ovarian carcinoma cell lines: new expression of cytokeratin 20.

**DOI:** 10.1038/bjc.1997.471

**Published:** 1997

**Authors:** T. Yanagibashi, I. Gorai, T. Nakazawa, E. Miyagi, F. Hirahara, H. Kitamura, H. Minaguchi

**Affiliations:** Department of Obstetrics and Gynecology, Yokohama City University School of Medicine, Yokohama, Japan.

## Abstract

**Images:**


British Journal of Cancer (1997) 76(7), 829–835
© 1997 Cancer Research Campaign

### Complexity of expression of the intermediate filaments of six new human ovarian carcinoma cell lines: new expression of cytokeratin 20

**T Yanagibashi[1], I Gorai[1], T Nakazawa[1], E Miyagi[1], F Hirahara[1], H Kitamura[2] and H Minaguchi[1]**

Departments of [1]Obstetrics and Gynecology and [2]Pathology, Yokohama City University School of Medicine, 3–9 Fukuura, Kanazawa-ku, Yokohama 236, Japan

**Summary** Six permanent human ovarian carcinoma cell lines (OVISE, OVTOKO, OVMANA and OVSAYO from clear cell adenocarcinoma, and OVSAHO and OVKATE from serous papillary adenocarcinoma) were established from solid tumours. The cell lines have been in culture for 5–8 years, the passage number varying from 62 to 246. Immunohistochemical analysis has shown that five of the six cell lines express at least six cytokeratin (CK) polypeptides. OVISE and OVSAYO expressed CKs 6, 7, 8, 18, 19 and 15 and/or 16. OVTOKO was positive for CKs 7, 8, 18, 19 and 15 and/or 16. OVSAHO expressed CKs 6, 7, 8, 14, 18, 19 and 15 and/or 16. OVMANA expressed CKs 6, 7, 8, 18, 19, 20 and 15 and/or 16. OVKATE expressed CKs 6, 7, 8, 13, 17, 18, 19, 20 and 15 and/or 16. The expression of CK7, additional expression of vimentin, and clinical and histopathological findings enabled us to confirm that six cell lines had been established from primary ovarian cancers. Two of the six cell lines were positive for CK20, although CK20 was not expressed in the original tumours. The heterotransplanted tumours produced by CK20-positive cells also expressed CK20. This is the first report of ovarian carcinoma cell lines that express CK20 irrespective of their histological type. CK20 has been found in all colon carcinoma cell lines, but only in the mucinous type of ovarian tumours. These new ovarian carcinoma cell lines will therefore provide a relevant experimental system for elucidating the regulatory control mechanisms of intermediate filament expression.

**Keywords:** human ovarian cancer; cell line; solid tumour; intermediate filament; cytokeratin 20 expression

The characteristics of ovarian cancer have been studied in primary tumours and in established cell lines that provide a reproducible source of tumour material (Buick et al, 1985; Horowitz et al, 1985; Hill et al, 1987; Sheer et al, 1987; Wolf et al, 1987; Langdon et al, 1988; Niekerk et al, 1988; Crickard et al, 1989; Hills et al, 1989; Wong et al, 1990; Möbus et al, 1992; Fuchtner et al, 1993; Morimitsu et al, 1993; Van Den Berg-Bakker et al, 1993). The characterization of relevant ovarian carcinoma cell lines is considered to be worthwhile for the investigation of their epithelial nature and derivation, and to demonstrate no contamination with fibroblasts. There have been only a few studies on the expression of intermediate filament (IF) in ovarian cancer cell lines. The cytokeratins (CKs) are known to be composed of a family of at least 20 different polypeptides and to be distributed in both normal and neoplastic tissues in a tissue-specific manner (Moll et al, 1982). Epithelial ovarian tumours are thought to arise from the ovarian surface epithelium or mesothelium (Scully, 1979; Van Niekerk et al, 1993). This hypothesis was supported by the presence of the so-called simple CKs 8 and 18 and, in greater amounts, CKs 7 and 19 (Moll et al, 1991). As most ovarian and colon carcinoma cell lines are derived from adenocarcinomas, differential diagnosis may sometimes be difficult on the basis of the intraoperative situation and the histopathological examination. The difference in the relative expression of CKs 7 and 20 was shown to be helpful in the distinction between primary ovarian carcinomas and metastatic adenocarcinomas of the gastrointestinal tract to the ovary (Wauters et al, 1995; Berezowski et al, 1996). CK20 has been found throughout all colon carcinoma cell lines and has been described as being the best marker for discriminating between adenocarcinoma cell lines derived from ovarian carcinomas and those derived from colon tumours (Möbus et al, 1994). The present study was aimed at immunohistochemically analysing IF expression, including that of CKs 7 and 20, in six newly established ovarian carcinoma cell lines that were derived from solid tumours. This study provides preliminary evidence for the expression of CK20 in these ovarian carcinoma cell lines, irrespective of their histological type.

*Received 24 June 1996*
*Revised 5 February 1997*
*Accepted 18 March 1997*

*Correspondence to:* I Gorai

### MATERIALS AND METHODS

### Cell lines

All the cell lines used were established from primary or metastatic solid tumours of human ovarian cancer. Four cell lines, OVISE, OVTOKO, OVMANA and OVSAYO, were derived from clear-cell adenocarcinoma and the other two, OVSAHO and OVKATE, were from serous papillary adenocarcinomas. The growth characteristics of the six cell lines are summarized in Table 1. Each metastatic tumour, from which OVISE, OVTOKO, OVSAHO or OVKATE were derived, was histologically identical to the original tumour.

### Morphological findings

The original tumour tissues, cultured on Lab Tek tissue chamber slides (Nunc, Naperville, IL, USA) at different passages, and xenografted tissues were fixed with 10% formaldehyde solution

Table 1 Clinical and biological characteristics of the cell lines

| Cell line | Initiation of culture | Source | Histopathology | Pretreatment | Tumour-associated antigen in serum |
|---|---|---|---|---|---|
| OVISE | 1988.10 | Solid metastatic | Clear cell adenocarcinoma | Six courses of CAP | CA125, IAP |
| OVTOKO | 1990.7 | Solid metastatic | Clear cell adenocarcinoma | Five courses of CAP | CA125, IAP TPA |
| OVSAHO | 1991.1 | Solid metastatic | Serous papillary adenocarcinoma | Fifteen courses of FAMT Six courses of CAP | CA125 CA72-4 |
| OVMANA | 1991.1 | Solid primary | Clear cell adenocarcinoma | Three courses of intraperitoneal cisplatin | CA125, CA19-9 CA72-4, IAP, TPA |
| OVSAYO | 1991.4 | Solid primary | Clear cell adenocarcinoma | No | CA19-9, TPA |
| OVKATE | 1991.12 | Solid metastatic | Serous papillary adenocarcinoma | Three courses of CAP Three courses of EP | CA125, TPA |

| Survival of patients (months) | Median doubling time (h) | Cell density (cells cm$^{-2}$) | CFE (%) | Tumorigenesis in nude mice s.c. | i.p. | Current passage |
|---|---|---|---|---|---|---|
| 36 | 60 | $6.0 \times 10^4$ | 36.0 | Yes | No | 246 |
| 2 | 70 | $6.0 \times 10^4$ | 45.0 | Yes | Yes | 186 |
| 7 | 56 | $2.7 \times 10^5$ | 4.2 | Yes | Yes | 112 |
| 1 | 67 | $1.9 \times 10^5$ | ND | Yes | No | 121 |
| Alive | 88 | $2.9 \times 10^5$ | 27.2 | No | No | 124 |
| 3 | 96 | $4.6 \times 10^5$ | 0.3 | Yes | Yes | 80 |

and stained with haematoxylin and eosin and periodic acid–Schiff. The morphology of ten slides of histological sections and tissue cultures and five slides of xenografted tissue sections were examined with the aid of a light microscope.

### Heterotransplantation in nude mice

Approximately $5 \times 10^6$ suspended cells were transplanted subcutaneously (s.c.) or intraperitoneally (i.p.) into Balb/c nude mice (6-week-old male mice; Shizuoka Experimented Animal Co., Shizuoka, Japan). The tumours were examined histologically.

### Antisera

The following monoclonal or polyclonal antibodies were used: (1) MAb OV-TL12/30, specific for CK 7; (2) MAb 35BH11, raised against CK8; (3) MAb DE-K13, raised against CK10 and CK13; (4) MAb MNF116, raised against CKs 10, 17 and 18; (5) MAb $E_3$, raised against CK17; (6) MAb DC 10, raised against CK18; (7) MAb RCK108, raised against CK19; (8) MAb BA17, raised against CK19; (9) MAb Ks20.8, raised against CK20; (10) MAb V9, anti-vimentin; (11) MAb D33, anti-desmin; (12) MAb E29 (1), anti-epithelial membrane antigen (EMA); (13) anti-S-100 protein; (14) anti-human myoglobin; (15) MAb 1A4, anti-human α-smooth muscle actin; (16) MAb 6F2, anti-human glial fibrillary acidic protein; (17) MAb EGFR1, anti-EGF receptor; (18) MAb K8.12, raised against CKs 13, 15 and 16; (19) MAb CY-90, raised against CK18; (20) MAb VIM 13.2, anti-vimentin; (21) anti-type II collagen; (22) MAb 528, anti-EGF receptor; (23) MAb Ks6,KA12, raised against CK6; (24) MAb IT-Ks20.3, raised against CK20; (25) MAb IT-Ks20.5, raised against CK20; (26) MAb IT-Ks20.10, raised against CK20; (27) MAb DE-K10, raised against CK10; (28) MAb LLOO2, raised against CK14; (29) MAb CYFRA, raised against CK19. The biotinylated goat anti-mouse immunoglobulin G, goat anti-rabbit immunoglobulin G and avidin-conjugated horseradish peroxidase were purchased from Nichirei Co. (Seikagaku Kohgyo Co., Tokyo, Japan).

Antisera (1)–(17) were purchased from Dakopatts (Dako Japan, Kyoto, Japan); (18)–(20) were purchased from Sigma (Sigma-Aldrich Co., Tokyo, Japan); (21) was purchased from Chemical International (Funakoshi Co., Tokyo, Japan); (22) was purchased from Oncogene Science (Cosmo Bio Co., Tokyo, Japan); (23)–(26) were purchased from Progen Biotechnik (Heidelberg, Germany); and (27) and (28) were purchased from Biomeda (Foster City, CA, USA). Antiserum (29) was kindly provided by Boehringer Mannheim (Tutzing, Germany).

### Immunohistochemical procedure

### *Single labelling procedure*

The avidin–biotin peroxidase complex method described by Gorai et al (1993) was used for single antigen localization. The single labelling technique was performed at four different passages. Cells cultured on coverslips (18 × 24 mm) were rinsed with phosphate-buffered saline (PBS) and then fixed by immersion first in 70% and then in 100% ethanol for 15 min each at –20°C, and then dried. The coverslips were incubated for 30 min at room temperature with non-immune mouse or rabbit serum before staining with one of the 10- to 1000-fold diluted primary antibodies for 30 min. The coverslips were washed with PBS and then incubated for 30 min with secondary antibody. After additional washing with PBS, the samples were incubated with avidin-conjugated horseradish peroxidase for 30 min. The coverslips were then washed with PBS and reacted with a substrate containing 3,3-diaminobenzidine for 5 min. After they were washed in PBS, the sections were dipped ten times into distilled water and then reacted with 0.5% copper sulphate in normal saline for 5 min. After being dipped several times in distilled water, counterstaining was performed with haematoxylin. The slides were dehydrated in xylene and embedded in synthetic resin.

 © Cancer Research Campaign 1997

An immunohistochemical study of the original ovarian tumours and heterotransplanted tumours was performed on formalin-fixed, paraffin-embedded sections using the avidin–biotin–peroxidase method, as described above. One representative block of each case was deparaffinized and rehydrated in graded alcohols. These sections were incubated in 0.01 M citrate buffer at 95°C for 60 min. The endogenous peroxidase activity was blocked with 0.3% hydrogen peroxide in methanol. The primary antibody was applied for 60 min and was followed by incubation with secondary antibodies for 10 min, and then with an avidin–biotin–peroxidase complex reagent for 10 min at room temperature. Sections were thoroughly washed with PBS between steps. Sections were reacted by immersion in Tris buffer containing 3,3-diaminobenzidine for 5 min.

The staining of the positive control slides (ovarian carcinomas for CK7 and colon carcinomas for CK20) was always positive and that of the negative control slides was negative at each staining. The expression of the antigens is defined as: –, negative; +, < 10% of cells positive; ++, 10–50% of cells positive; +++, > 50% of cells positive.

### *Double labelling procedure*

For double labelling studies of CKs 7 and 20, streptavidin–biotin labelling (Giorno, 1984) and alkaline phosphatase monoclonal anti-alkaline phosphatase procedures (Malik and Daymon, 1982; Cordell et al, 1984) were performed sequentially. The double labelling technique was performed at three different passages. After fixing and drying, the samples were first treated with normal goat serum for 5 min at room temperature. The coverslips were then incubated with one of the primary antisera, diluted 50- to 500-fold with PBS, for 20 min and then washed with PBS. The samples were reacted with a biotinylated secondary goat anti-mouse antibody for 10 min and then washed with Tris buffer. The coverslips were incubated with horseradish peroxidase-labelled streptavidin for 10 min, washed with Tris buffer and then reacted with 4-chloronaphthol for 20 min, giving a blue end product. After they were washed with PBS, the samples were incubated with 0.1 M glycine chloride, pH 2.2, for 2 h and then washed again with PBS. In the second alkaline phosphatase–anti-alkaline phosphatase procedure, the samples were first incubated with a second primary antiserum overnight at 4°C and washed with Tris buffer. The coverslips were reacted with secondary rabbit anti-mouse antibody for 30 min and then washed with Tris buffer. The samples were reacted with mouse alkaline phosphatase–anti-alkaline phosphatase immune complex for 30 min and then washed with Tris buffer. The coverslips were then reacted with substrates of naphthol AS-MX phosphate and Fast Red TR for 20 min, giving a red end product. After counterstaining, the samples were embedded in glycerin. For staining of the original tumours and heterotransplanted tumours, sections were prepared and reacted in the same way as described for the single labelling procedure.

### RESULTS

### Heterotransplantation

The OVISE, OVTOKO, OVSAHO, OVMANA and OVKATE cells produced 1.0-cm- to 1.5-cm-diameter tumours in the subcutis of male Balb/c nude mice from 2–3 months after s.c. transplantation. Histological examination of the tumours showed a structure of sheets

**Table 2** Expression of intermediate filaments and other proteins in ovarian cancer cell lines

| Cell lines | Cytokeratins | | | | | | | |
|---|---|---|---|---|---|---|---|---|
| | Ks6, KA12 CK6 | OV-TL12/30 CK7 | 35BH11 CK8 | DE-K10 CK10 | DE-K13 CK10 and 13 | MNF116 CK10, 17 and 18 | K8, 12 CK13, 15 and 16 | LL002 CK14 |
| OVISE | + | +++ | ++ | – | – | +++ | +++ | – |
| OVTOKO | – | + | ++ | – | – | +++ | +++ | – |
| OVSAHO | + | ++ | +++ | – | – | +++ | +++ | + |
| OVMANA | + | +++ | ++ | – | – | +++ | +++ | – |
| OVSAYO | + | +++ | +++ | – | – | +++ | +++ | – |
| OVKATE | ++ | +++ | + | – | + | +++ | +++ | – |

| Cell lines | E3 CK17 | DC10 CK18 | CY-90 CK18 | RCK108 CK19 | BA17 CK19 | CYFRA CK19 | IT-Ks20.3 CK20 | IT-Ks20.5 CK20 | IT-Ks20.10 CK20 | Vimentin V9 | Vimentin VIM13.2 | Desmin |
|---|---|---|---|---|---|---|---|---|---|---|---|---|
| OVISE | – | +++ | +++ | +++ | +++ | + | – | – | – | + | ++ | – |
| OVTOKO | – | ++ | +++ | + | – | + | – | – | – | ++ | +++ | – |
| OVSAHO | – | +++ | +++ | + | – | + | – | – | – | +++ | +++ | – |
| OVMANA | – | ++ | +++ | +++ | ++ | + | – | – | + | +++ | ++ | – |
| OVSAYO | – | + | +++ | +++ | ++ | + | – | – | – | + | ++ | – |
| OVKATE | +++ | + | +++ | +++ | +++ | + | – | ++ | – | + | + | – |

| Cell lines | EMA | S-100 | Myoglobin | Collagen (type II) | α-Smooth muscle actin | Glial fibrillary acidic protein | EGF-R 528 | EGF-R EGFRI (1) |
|---|---|---|---|---|---|---|---|---|
| OVISE | +++ | – | – | – | – | – | ++ | ++ |
| OVTOKO | + | – | – | – | – | – | +++ | ++ |
| OVSAHO | ++ | – | – | – | – | – | + | – |
| OVMANA | +++ | – | – | – | – | – | + | – |
| OVSAYO | ++ | – | – | – | – | – | +++ | +++ |
| OVKATE | ++ | – | – | – | – | – | +++ | ++ |

As determined by immunohistochemistry: –, negative; +, < 10% of cells positive; ++, 10–50% of cells positive; +++, > 50% of cells positive.

© Cancer Research Campaign 1997

**Figure 1** Expression of cytokeratin 7 (arrows) (**A**) and 20 (arrows) (**B**) on OVMANA cells. Scale bar = 100 µm

**Figure 2** Expression of cytokeratin 20 on heterotransplanted tumour (arrows) produced by OVKATE cells. Scale bar = 100 µm of tumour cells with clear cytoplasm (OVISE), a tubular structure of tumour cells with clear eosinophilic cytoplasm (OVTOKO), sheets or papillary structures of oval- to spindle-shaped atypical cells (OVSAHO), atypical glandular structures lined with round cells with clear or light cytoplasm (OVMANA) and solid nests or papillary structures of oval- to spindle-shaped cells (OVKATE), which resembled the original tumours from which the cell lines were derived. The OVSAYO cells did not produce any s.c. tumours. Intraperitoneal transplantation of OVTOKO, OVSAHO and OVKATE cells produced metastatic tumours and/or dissemination in the peritoneal cavity.

**Figure 3** Coexpression of cytokeratin 7 and 20 on OVMANA cells: blue colour stains cytokeratin 7 (arrows) and red colour stains cytokeratin 20 (arrows). Scale bar = 50 µm

**Table 3** Expression of intermediate filaments in original ovarian tumours

| | Cytokeratins | | | | | |
|---|---|---|---|---|---|---|
| **Original tumours** | **OV-TL12/30 CK7** | **35BH11 CK8** | **CY-90 CK18** | **RCK108 CK19** | **Ks20.8 CK20** | **IT-Ks20.10 CK20** |
| OVISE | +++ | + | ++ | +++ | – | – |
| OVTOKO | +++ | – | – | – | – | – |
| OVSAHO | + | + | + | + | – | – |
| OVMANA | + | – | ++ | + | – | – |
| OVSAYO | + | ++ | – | – | – | – |
| OVKATE | +++ | ++ | + | + | – | – |

As determined by immunohistochemistry: –, negative; +, < 10% of cells positive; ++, 10–50% of cells positive; +++, > 50% of cells positive.

**Table 4** Expression of intermediate filaments in heterotransplanted tumours

| | Cytokeratins | | | | | |
|---|---|---|---|---|---|---|
| **Hetero-transplanted tumours** | **OV-TL12/30 CK7** | **35BH11 CK8** | **CY-90 CK18** | **RCK108 CY19** | **Ks20.8 CK20** | **IT-Ks20.10 CK20** |
| OVISE | ++ | + | + | ++ | – | – |
| OVTOKO | +++ | – | – | – | – | – |
| ONSAHO | + | – | + | – | – | – |
| OVMANA | ++ | ++ | ++ | ++ | + | + |
| OVSAYO | NT | NT | NT | NT | NT | NT |
| OVKATE | +++ | + | ++ | + | + | + |

As determined by immunohistochemistry: –, negative; +, < 10% of cells positive; ++, 10–50% of cells positive; +++, > 50% of cells positive. NT not tested.

© Cancer Research Campaign 1997

### Patterns of intermediate filaments and other proteins

#### *Cell lines*

The six cell lines yielded various patterns of CK expression, although the expressions of vimentin, desmin, EMA and other proteins were identical in all of the cells (Table 2). The OVISE and OVSAYO cells were positive for CKs 6, 7, 8, 18, 19 and 15 and/or 16 and were negative for CKs 10, 13, 14, 17 and 20. The OVTOKO cells expressed CKs 7, 8, 18, 19 and 15 and/or 16 and were negative for CKs 6, 10, 13, 14, 17 and 20. The OVSAHO cells showed a reactivity to CKs 6, 7, 8, 14, 18, 19 and 15 and/or 16 and no reactivity to CKs 10, 13, 17 and 20. The OVMANA cells were positive for CKs 6, 7, 8, 18, 19, 20 and 15 and/or 16, and were negative for CKs 10, 13, 14 and 17 (Figure 1). The OVKATE cells expressed CKs 6, 7, 8, 13, 17, 18, 19, 20 and 15 and/or 16, and were negative for CKs 10 and 14. All the cells were positive for vimentin, EMA and EGF receptors and were negative for desmin, S-100, myoglobin, type II collagen, α-smooth muscle actin and glial fibillary acidic protein. Positive staining for each antigen in the six cell lines was identical at both early (10th passage to 14th passage) and late (70th passage to 116th passage) passages.

#### *Original tumours*

The original tumours yielded various patterns of CK expression. All the tumours were positive for CK7 and negative for CK20. The tumours of colon carcinoma expressed CK20. The tumours from which OVISE, OVSAHO and OVKATE were established expressed CKs 8, 18 and 19, whereas the tumour from which OVTOKO was established was negative for these particular CKs (Table 3).

#### *Heterotransplanted tumour*

The CK expression of the heterotransplanted tumours was similar to that of the original tumours except for that of CK20. All the tumours were positive for CK7, whereas the tumours consisting of CK20-positive OVMANA and OVKATE cells were also positive for CK20 (Figure 2 and Table 4).

#### *Double labelling*

The double labelling technique showed that OVMANA and OVKATE cell lines were composed of four types of cells: CK7-positive, CK20-positive; CK7-positive, CK20-negative; CK7-negative, CK20-positive; and CK7-negative, CK20-negative cells (Figure 3). Furthermore, these four types of cells were observed in the heterotransplanted tumours produced by OVMANA and OVKATE cells. In contrast, two types of cell, CK7-positive, CK20-negative, and CK7-negative, CK20-negative, were also observed in the original ovarian tumours from which OVMANA and OVKATE cells were derived.

### DISCUSSION

Different human epithelium-derived cell lines have been shown to produce tonofilaments containing different sets of CK polypeptides (Franke et al, 1982). Most cell lines express three or four CK polypeptides, including components 8 and 18. Some cell lines (for example MCF-7 and HT-29) produce the small and acidic CK19, which has been identified in some cell cultures (Fuchs and Green, 1981; Wu and Rheinwald, 1981). The most complex expression of CK polypeptides has been observed in the cell line A-431, which is derived from an epidermoid carcinoma of the vulva. The clonal sublines of A-431 cell line continued to express as many as ten different CK polypeptides (Moll et al, 1982). Five of the six cell lines examined in the present study expressed at least six CK polypeptides. OVKATE cells retained the ability to express as many as nine CK proteins. This might reflect the multipotentiality of the differentiation of Müllerian duct-derived epithelia (Moll et al, 1991).

CK20 has only recently been introduced as a new CK polypeptide (Moll et al, 1990). CK20 expression is almost entirely confined to the mucosa of the small and large intestine, the gastric foveolar epithelium, the umbrella cells of the urothelium and epidermal Merkel cells (Moll et al, 1992). This specificity is maintained in the corresponding tumours. In an immunohistochemical study, 89 out of 92 cases of colorectal adenocarcinomas expressed CK20, whereas 31 out of 34 serous, endometrioid, anaplastic and clear cell ovarian carcinomas were completely negative; the remaining three were essentially negative for CK20 (Moll et al, 1992). CK20 has been found in all colon carcinoma cell lines, but not in all ovarian carcinoma cell lines (Möbus et al, 1994). CK20 was effective in discriminating between metastatic and primary ovarian carcinomas. The only exception was a group of mucinous ovarian tumours (both adenomas and carcinomas), which consistently expressed CK20, irrespective of their degree of malignancy (Moll et al, 1992). This sometimes made it difficult to differentiate between primary and metastatic mucinous adenocarcinomas in the ovary.

In the study reported here (Table 2), CK20 was expressed in one clear-cell adenocarcinoma cell line and one serous papillary adenocarcinoma cell line. To the best of our knowledge, this is the first report that demonstrates the expression of CK20 in ovarian carcinoma cell lines without regard to their histological type. In addition, CK20-positive cell lines expressed CK7 and vimentin. It has been shown that the expression of simple epithelial CK7 occurs only in ovarian carcinoma cell lines and is completely absent from colon carcinoma cell lines (Ramaekers et al, 1990; Ueda et al, 1993; Möbus et al, 1994). However, CK7 staining was found in ovarian metastatic tumours from primary gastric and colonic adenocarcinomas (Wauters et al, 1995). An additional differential marker is vimentin, which has been shown to be strongly expressed in the ovarian carcinoma cell lines, but which is essentially absent from the colon carcinoma cell lines (Viale et al, 1988; Coggi et al, 1989; Möbus et al, 1994; Gorai et al, 1995). The tumours deriving CK20-positive cell lines have been considered to be primary ovarian carcinoma, thus excluding the possibility of metastasis of colon cancer to the ovary on the basis of clinical and histopathological findings, and a critical review of the original histology by at least two independent pathologists.

CK20, however, was not expressed in the original ovarian tumours from which the CK20-positive OVMANA and OVKATE cell lines were derived, although CK20 was positive for colon carcinomas. CK20 was expressed in the heterotransplanted tumours produced by the CK20-expressing cell lines. It has been reported that patterns of CK expression in cultured cells are not decisively correlated with the CK present in the corresponding tissue (Fuchs and Green, 1978, 1981; Sun and Green, 1978; Doran et al, 1980; Wu and Rheinwald, 1981). The fact that CK20 was newly expressed in ovarian carcinoma cells other than mucinous tumours is of clinical importance when we consider the origin of CK20-positive cells. It is possible that the pattern of expression is altered during the establishment of cell lines. The regulatory

© Cancer Research Campaign 1997

control mechanisms of CK20 expression in ovarian carcinoma cell lines from CK20-negative ovarian tissues remain to be elucidated. First, it may be that the loss of control mechanisms in individual cells occurs, similar to the case for CKs 8 and 18 (Knapp and Franke, 1989). In SV40-transformed fibroblasts, the CK18 gene was constitutively transcribed into translatable mRNA but the protein was rapidly degraded in the absence of its complex partner, CK8. Secondly, under certain conditions external environmental factors may play an important role in modulating epithelial differentiation. Cultured rabbit skin, corneal and oesophageal epithelial cells have been shown to express more keratin proteins than were originally present in intact tissues or observed in cysts formed by cultured cells in athymic mice (Doran et al, 1980). Thirdly, a new subclone arises from the descendants of the original transformed cell and, with progressive growth in vitro, the cell population becomes enriched with those variants that produce CK20 (Tannock, 1987). Some cholangiocarcinomas of the liver have demonstrated a positive reactivity for CK20, whereas all non-neoplastic bile ducts and ductules were negative, suggesting a correlation between the CK20 expression and neoplastic transformation of the bile duct (Maeda et al, 1996).

In summary, six newly established human ovarian carcinoma cell lines that were derived from solid tumours have been investigated in terms of their immunohistochemical properties, including IF expression. All six cell lines were positive for CKs 7, 8, 18 and 19. In the past, the expression of CK20 has been considered to be entirely confined to the mucosa of the small and large intestine. Although CK20 was not expressed in the original ovarian tumours, two of the six cell lines (one clear-cell adenocarcinoma and one serous papillary adenocarcinoma) expressed CK20, as did the heterotransplanted tumours produced by those CK20-positive cell lines. These cell lines will provide a relevant experimental system for assessing the regulatory control mechanisms of IF expression, including a family of CKs.

### ACKNOWLEDGEMENTS

The authors thank Mrs Atsuko Fukui and Mrs Kumiko Tanaka for technical assistance. We are also grateful to Mrs Michiko Kogure for preparing the manuscript.

### REFERENCES

Berezowski K, Stastny JF and Kornstein MJ (1996) Cytokeratins 7 and 20 and carcinoembryonic antigens in ovarian and colonic carcinoma. *Mod Pathol* **9**: 426–429

Buick RN, Polland R and Trent JM (1985) Comparative properties of five human ovarian adenocarcinoma cell lines. *Cancer Res* **45**: 3668–3676

Coggi G, Dell Orto P and Braidotti P (1989) Coexpression of intermediate filaments in normal and neoplastic human tissues: a reappraisal. *Ultrastruct Pathol* **13**: 501–514

Cordell JL, Faini B, Erber WN, Ghosh AK, Abdulaiz Z, MacDonald S, Fulford KAF, Stein H and Mason DY (1984) Immunoenzymatic labeling of monoclonal antibodies using immune complexes of alkaline phosphatase and monoclonal anti-alkaline phosphatase (APAAP complexes). *J Histochem Cytochem* **32**: 219–229

Crickard K, Niedbala MJ, Crickard U, Yooness M, Sandberg AA, Okuyama K, Bernaki RJ and Sathidanand SK (1989) Characterization of human ovarian and endometrial carcinoma cell lines established on extracellular matrix. *Gynecol Oncol* **32**: 163–173

Doran TI, Vidrich A and Sun T-T (1980) Intrinsic and extrinsic regulation of the differentiation of skin, corneal and esophageal epithelial cells. *Cell* **22**: 17–25

Franke WW, Schmid E and Moll R (1982) The intermediate filament cytoskeleton in tissues and in cultured cells: differentiation specificity of expression of cell architectural elements. In *Human Carcinogenesis*, Harris CC and Autrup HN (eds), pp. 3–33. Academic Press: New York

Fuchtner C, Emma DA, Manetta A, Gamboa G, Bernstein R and Liao SY (1993) Characterization of a human ovarian carcinoma cell line: UCI 101. *Gynecol Oncol* **48**: 203–209

Fuchs E and Green H (1978) The expression of keratin genes in epidermis and cultured epidermal cells. *Cell* **15**: 887–897

Fuchs E and Green H (1981) Regulation of terminal differentiation of cultured human keratinocytes by vitamin A. *Cell* **25**: 617–625

Giorno R (1984) A comparison of two immunoperoxidase staining methods based on the avidin–biotin interaction. *Diagn Immunol* **2**: 161–166

Gorai I, Doi C and Minaguchi H (1993) Establishment and characterization of carcinoma cell line of the human uterus. *Cancer* **71**: 775–786

Gorai I, Nakazawa T, Miyagi E, Hirahara F, Nagashima Y and Minaguchi H (1995) Establishment and characterization of two human ovarian clear cell adenocarcinoma lines from metastatic lesions with different properties. *Gynecol Oncol* **57**: 33–46

Hill BT, Whelan RDH, Gibby EM, Sheer D, Hosking LK, Shellard SA and Rupniak T (1987) Establishment and characterization of three new human ovarian carcinoma cell lines and initial evaluation of their potential in experimental chemotherapy studies. *Int J Cancer* **39**: 219–225

Hills CA, Kelland LR, Abel G, Siracky J, Wilson AP and Harrap KR (1989) Biological properties of ten human ovarian carcinoma cell lines: calibration in vitro against four platinum complexes. *B J Cancer* **59**: 527–534

Horowitz AT, Treves AJ, Voss R, Okon E, Fuks Z, Davidson L and Biran S (1985) A new human ovarian carcinoma cell line: establishment and analysis of tumor-associated markers. *Oncology* **42**: 332–337

Knapp AC and Franke WW (1989) Spontaneous losses of control of cytokeratin gene expression in transformed, non-epithelial human cells occurring at different levels of regulation. *Cell* **59**: 67–79

Langdon SP, Lawrie SS, Hay FG, Hawkes MM, McDonald A, Hayeard IP, Schol DJ, Higers J, Leonald RC and Smyth JF (1988) Characterization and properties of nine human ovarian adenocarcinoma cell lines. *Cancer Res* **48**: 6166–6172

Maeda T, Kajiyama K, Adachi E, Takenaka K, Sugimachi K and Tsuneyoshi M (1996) The expression of cytokeratins 7, 19 and 20 in primary and metastatic carcinomas of the liver. *Mod Pathol* **9**: 901–909

Malik NJ and Daymon ME (1982) Improved double immunoenzymatic labeling using alkaline phosphatase and horseradish peroxidase. *J Clin Pathol* **35**: 1092–1094

Möbus V, Gerharz CD, Press U, Moll R, Beck T, Mellin W, Pollow K, Knapstein PG and Kreienberg R (1992) Morphological, immunohistochemical and biological characterization of 6 newly established human ovarian carcinoma cell lines. *Int J Cancer* **52**: 76–84

Möbus VJ, Moll R, Gerharz CD, Kieback DG, Weikel W, Hoffmann G and Kreienberg R (1994) Establishment of new ovarian and colon carcinoma cell lines: differentiation is only possible by cytokeratin analysis. *Br J Cancer* **69**: 422–428

Moll P, Pitz S, Levy R, Weikel W, Franke WW and Czernobilsky B (1991) Complexity of expression of intermediate filament proteins, including glial filament protein, in endometrial and ovarian adenocarcinomas. *Hum Pathol* **22**: 989–1001

Moll R, Franke WW, Schiller DL, Geiger B and Krepler R (1982) The catalog of human cytokeratins: patterns of expression in normal epithelia, tumors and cultured cells. *Cell* **31**: 11–24

Moll R, Schiller DL and Franke WW (1990) Identification of protein IT of the intestinal cytokeratin as a novel type I cytokeratin with unusual properties and expression patterns. *J Cell Biol* **111**: 567–580

Moll R, Löwe A, Laufer J and Franke WW (1992) Cytokeratin 20 in human carcinomas. A new histodiagnostic marker detected by monoclonal antibodies. *Am J Pathol* **140**: 427–447

Morimitsu Y, Yano H and Kojiro M (1993) Morphologic characteristics, proliferation, and tumor marker expression of two human ovarian carcinoma cell lines in three dimensional culture. *Gynecol Oncol* **48**: 155–164

Niekerk CC, Van Poels LG, Jap PHK, Smeets DFCM, Thomas CMG, Ramaekers FCS and Vooijs GP (1988) Characterization of a human ovarian carcinoma cell line, OTN 14, derived from a mucinous cytoadenocarcinoma. *Int J Cancer* **42**: 104–111

Ramaekers F, Van Niekerk C, Poels L, Schaatsma E, Huijsmans A, Robben H, Schaart G and Vooijs P (1990) Use of monoclonal antibodies to keratin 7 in the differential diagnosis of adenocarcinomas. *Am J Pathol* **136**: 641–655

Scully RE (1979) Tumours of the ovary and maldeveloped gonads. In *Atlas of Tumor Pathology*, Second series fascicle 16, Hartoman WH eds, Armed Forces Institute of Pathology: Washington, DC

© Cancer Research Campaign 1997

Sheer D, Sheppard DM, Gorman PA, Ward B, Whelan RD and Hill BT (1987) Cytogenetic analysis of four human ovarian carcinoma cell lines. *Cancer Genet Cytogenet* **26**: 339–349

Sun T-T and Green H (1978) Keratin filaments of cultured human epidermal cells. Formation of intermolecular disulfide bonds during terminal differentiation. *J Biol Chem* **253**: 2053–2060

Tannock IF (1987) Tumor growth and cell kinetics. In *The Basic Science of Oncology*, Tannock IF and Hill RP (eds), p.140. Pergamon Press: New York

Ueda G, Sawada M, Ogawa H, Tanizawa O and Tsujimoto M (1993) Immunohistochemical study of cytokeratin 7 for the differential diagnosis of adenocarcinomas in the ovary. *Gynecol Oncol* **51**: 219–223

Van Den Berg-Bakker CAM, Hagemeijer A, Franken-Postma EM, Smit VTHB, Kuppen PJK, Van Ravenswaay Claasen HH, Corunelisse CJ and Schrier PI (1993) Establishment and characterization of 7 ovarian carcinoma cell lines and granulosa tumor cell lines: growth features and cytogenesis. *Int J Cancer* **53**: 613–620

Van Niekerk CC, Ramaekers FCS, Hanselaar AGJM, Aldewireldt J and Poels LG (1993) Changes in expression of differentiation markers between normal ovarian cells and derived tumors. *Am J Pathol* **142**: 157–177

Viale G, Gambacorta M, Dell'Orto P and Coggi G (1988) Coexpression of cytokeratins and vimentin in common epithelial tumors of the ovary: an immunocytochemical study of eighty-three cases. *Virchows Arch A (Pathol Anat)* **412**: 91–101

Wauetrs CCAP, Smedts F, Gerrits LGM, Bosman FT and Ramaekers FCS (1995) Keratins 7 and 20 as diagnostic markers of carcinomas metastatic to the ovary. *Hum Pathol* **26**: 852–855

Wolf CW, Hayward JP, Lawrie SS, Buckton K, McIntyre MA, Adams DJ, Lewis AD, Scott ARR and Smyth JF (1987) Cellular heterogeneity and drug resistance in two ovarian adenocarcinoma cell lines derived from a single patient. *Int J Cancer* **39**: 695–702

Wong WSF, Wong YF, Ng YTA, Huang PD, Chew EC, Ho TH and Chang MZA (1990) Establishment and characterization of a new human cell line derived from ovarian clear cell carcinoma. *Gynecol Oncol* **38**: 37–45

Wu Y-J and Rheinwald JG (1981) A new small (40 kd) keratin filament protein made by some cultured human squamous cell carcinomas. *Cell* **25**: 627–635

© Cancer Research Campaign 1997